# Establishing Interprofessional Medication Reviews in Home Care Patients – A Feasibility Study

**DOI:** 10.2147/DHPS.S519424

**Published:** 2025-10-08

**Authors:** Carla Meyer-Massetti, Anita Sommer, Estelle Kaiser, Anna Maria Peschak, Marina Preisig, Carole Elodie Aubert, Raphael Dimitri Mäder, Lisa Ramseier, Irene Stefanie Riesen, Stefanie Lettieri-Amstutz, Daniela Rölli, Nicole Schönenberger, Christoph R Meier

**Affiliations:** 1 Institute of Primary Health Care (BIHAM), University of Bern, Bern, Switzerland; 2 Clinical Pharmacy and Epidemiology, Department of Pharmaceutical Sciences, University of Basel, Basel, Switzerland; 3 Spitex Bern, Bern, Switzerland; 4 Spitex Stadt Luzern, Lucerne, Switzerland; 5 Department of General Internal Medicine, Inselspital, Bern University Hospital, Bern, Switzerland; 6 Clinical Pharmacology and Toxicology, Department of General Internal Medicine, Inselspital - University Hospital Bern, Bern, Switzerland; 7 Graduate School for Health Sciences, University of Bern, Bern, Switzerland; 8 Hospital Pharmacy, University Hospital of Basel, Basel, Switzerland

**Keywords:** medication safety, polypharmacy, potentially inappropriate medication, home care, interprofessional collaboration, medication review

## Abstract

**Background:**

Medication-related problems are especially frequent among home-care patients, who are predominantly older, multimorbid, polymedicated and interact with multiple professionals, necessitating timely, complete and accurate drug information communication.

**Aim:**

To pilot a standardised approach to interprofessional home-care medication management focusing on deprescribing.

**Methods:**

Home-care patients, cared for in their own homes by one professional nursing agency in the city of Bern, Switzerland, aged ≥64 and taking ≥4 prescribed medications were assessed for medication-related risk with the interprofessional, 10-item doMESTIC RISK tool. Community pharmacists performed structured medication reviews for at-risk patients, communicating with primary care physicians via standardised form.

**Results:**

Nurses initiated 106 risk analyses (as defined in the process) in consenting patients, with 76 (72%) completed by pharmacists; 30 analyses could not be completed due to missing information. Out of 76 patients with a completed risk assessment, 26 patients did not need a medication review; one score was missing. The 49 patients qualifying for a medication review were on average 84.0 ± 7.7 years old and took a mean of 11.2 ± 4.5 prescribed medications regularly and 2.8 ± 3.5 prescribed as-needed medications. Pharmacists identified a median of two problems per patient, suggesting 64 potential interventions. Forty-three priority interventions for 18 patients were communicated to physicians, mostly dose reduction (27%) and therapy cessation (23%). Despite providing comprehensive information in the requests, physicians only reacted to 50% of pharmacists’ recommendations (9/18 patients), accepting 57% of pharmacists suggestions in nine patients, predominantly deprescribing (nine medications).

**Conclusion:**

While pharmacists identified medication improvements through structured medication reviews, limited access to clinical information and insufficient communication between health care professionals were key barriers. Strengthening interprofessional collaboration and structured communication through shared platforms allowing clinical data exchange is essential to optimizing medication management in home-care. Clarification of the roles of the team members must be improved.

## Introduction

The medication use process causes 30–50% of adverse events in health care.^1^ To date, medication safety research has mainly focused on institutional settings, mainly in the United States. The transferability of these findings to Europe’s home-care sector might be limited by its different health care environment.

The existing literature indicates that up to 30% of home-care patients experience medication-related problems (MRPs), encompassing adverse drug events and medication errors.^2–4^ Home healthcare in Switzerland refers to a range of professional nursing and support services provided to people in their own homes, enabling them to live independently despite illness, disability, or age-related needs. The most common provider of home-healthcare is Spitex, a network of non-profit and private organizations whose name stands for “spitalexterne Hilfe und Pflege” (out-of-hospital help and care) (www.spitex.ch).

Older adult patients are a specifically vulnerable group due to their age and medication use.^3^,^5^ This has also been confirmed for patients receiving professional home-care by nurses.^6^ Overall, several studies have shown MRPs to be much more frequent in ambulatory-care settings than inpatient settings.^6^

Medication-use practices in home-care organizations are complex, involving multiple steps from preparation to administration, the participation of different professional groups, and frequent care transitions.^7^ Polypharmacy is highly prevalent: one study found that home-care patients were prescribed a mean of 7.5 ± 3.5 drugs per day.^7^ The *Helsana Arzneimittel Report 2020* by one of Switzerland’s major health assurance companies, analysed and published annual medication-use data and found even higher rates of polypharmacy—a mean of 16 medications per home-care patient per day.^8^ This pattern of old age, polypharmacy and multiple care interfaces increases the risk of MRPs such as potentially inappropriate medications, side effects and impaired adherence. Additionally, multimorbid, polymedicated patients often need several types of health care professionals, involving several care interfaces and communication challenges. This was also confirmed in a failure, mode and effects analysis that assessed error-prone process steps in home-care settings.^7^

A study published in 2018 emphasised the challenges of multiple interfaces when home-care patients were discharged from inpatient care, including the timely transfer of complete, accurate medication information, securing drug supply to patients’ homes and prescription quality.^9^ Communication issues between patients, family caregivers and health care professionals are central to these problems.

While previous studies showed a clear need to improve medication safety for home-care patients (6), little evidence was produced on adequate processes.

Within this study, we aimed to implement a structured, interprofessional process for home-care nurses, community pharmacists and primary care physicians (PCPs) executing medication reviews for older adult home-care patients with polypharmacy at risk of MRPs. Our focus was on medication safety improvement through deprescribing, including drug discontinuation, dose reduction and simplification.^10^

## Ethics Approval

The present study was performed in line with the principles of the Declaration of Helsinki. The Cantonal Ethics Committee of Bern decided that an ethics approval was unnecessary, the study falling into the category of quality improvement, not under the law on human research (ethics submission ID 2022–00517).

## Methods

This single-center, mixed-methods, quality improvement study was executed in the city of Bern, Switzerland, involving the *Spitex Bern* home-care organisation, 14 voluntarily participating community pharmacies and the PCPs of the participating home-care patients. The Institute for Primary Health Care BIHAM of the University of Bern reached 925 primary care providers via their newsletter, informing them about the study. The study sought to extend upon the pharmacist’s classic role as a prescription validator. An in-depth prescription validation took place in the shape of a medication review for patients who had been previously identified as being at risk of MRPs. The most significant findings from each medication review were reported on a structured form to the PCPs (see Supplementary File C). They took the final decisions and informed the community pharmacies and the home-care organisation. Data on medication changes decided by the physician were collected by the community pharmacists involved in the study.

### Study Participants

After being contacted by our researchers, the biggest home-care organisation in the city of Bern, *Spitex Bern*, agreed to participate in the study. *Spitex Bern* employs approximately 400 collaborators providing professional care in the city of Bern and the commune of Kehrsatz. Services include advice, basic care and treatments, and specialist services in dementia, psychiatry, palliative care and wound care. *Spitex Bern* is a not-for-profit organisation with a service mandate from the Canton of Bern.

Community pharmacies around Bern were contacted through their cantonal professional society and invited to participate in the study. If interested, they received a factsheet explaining the study and the tasks to be completed by the pharmacists. One follow-up request was sent by Email for motivational purposes, if no reply was received in four weeks. Inclusion was based on written consent.

This study focused on patients supported by *Spitex Bern*, and who were clients of one of the participating pharmacies. Patients were cared for in their own homes by *Spitex Bern.*

The inclusion criteria required patients to be aged ≥64, and taking ≥4 physician-prescribed medications, according to the population the doMESTIC RISK screening tool was developed for. In addition, patients had to be able to understand and read German (study materials were only available in German, the main language of the city of Bern), and giving written informed consent. Exclusion criterion, in addition to patient age >64 years and <4 physician prescribed medication, was the inability to sign informed consent, for example, because of dementia.

Patients could be enrolled via their community pharmacist or home-care nurse. Pharmacists and nurses were encouraged to proactively screen their patients for eligibility. Eligible patients were orally invited to participate as per the patient information sheet. If willing, they were asked to sign the informed consent form. No compensation was provided for study participation.

The University of Bern’s Institute of Primary Health Care and the Bern Physicians’ Association informed 925 PCPs about the project via an email-newsletter with an attached factsheet describing the study, asking them to participate if contacted by community pharmacies.

Due to the exploratory nature of this pilot study, no sample size calculation was possible.

### Participant Training

The research team trained participating pharmacists for their study tasks using one-hour sessions, either face-to-face or virtually. Topics covered were facts on medication safety in home-care, the use of the risk assessment tool, the execution of structured medication reviews based on the checklist provided, the use of the physician request form and data collection. The research team used face-to-face training sessions for the home-care nurses responsible for quality management within their teams. They subsequently educated their team members using a train-the-trainer approach.

Participating nurses and pharmacists received paper-based and electronic versions of the educational materials in German (available upon request from the authors). The research team was available via Email or telephone during the implementation phase. Physicians received a factsheet, detailing the study, via an email-newsletter.

### Implementation Process

**Figure 1:**
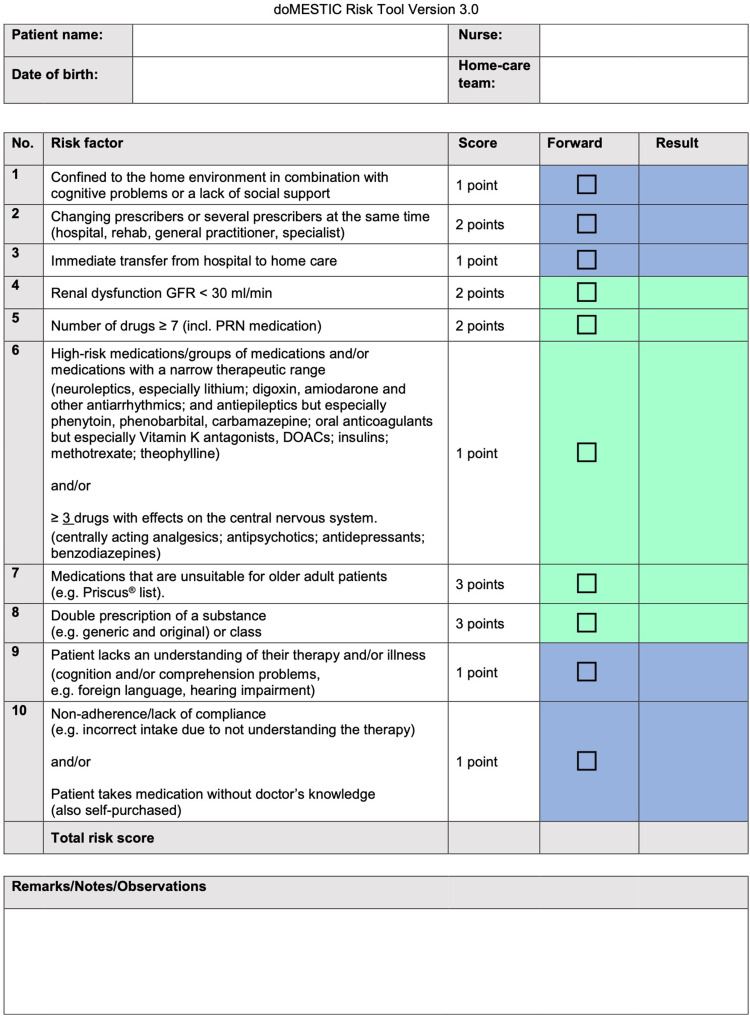
Interprofessional doMESTIC RISK assessment tool.^11^ Green: Assessment by the home-care nurse. Blue: Assessment by the community pharmacist. An assessment tool user manual in German is available from the authors upon request.

*Spitex Bern* screened its database for patients eligible for the study. If so, they informed their patients as per the patient information sheet and asked them to consent to participate. *Spitex Bern* nurses started to complete the doMESTIC RISK assessment tool^11^ for each participating patient. This was then forwarded via secure Email for completion by the patient’s community pharmacist. If the assessment score was <5, the pharmacist informed *Spitex Bern*, which in turn let the patient know that they were at a low risk of MRPs and would not be included in the study. The doMESTIC RISK assessment tool^11^ is shown in Figure 1, with detailed instructions in Supplementary File A.

The medication of patients with a risk score of ≥5 was reviewed in detail using a structured medication analysis checklist to assess and categorize medication-related problems (Supplementary File B).

Findings were prioritised in collaboration with the home-care nurse and the patient, if appropriate, and priority findings were presented to their PCP via a standardised form (Supplementary File C).

The PCP could respond to those medication suggestions on the same form and return it to the pharmacy together with a new prescription. *Spitex Bern* received an updated medication list.

**Figure 2:**
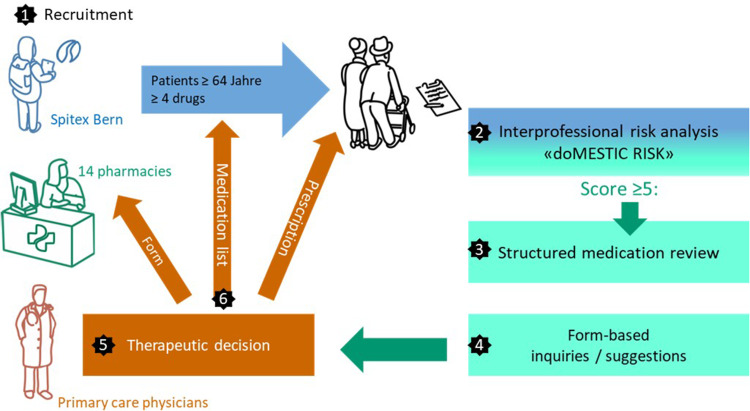
The study process.

The complete project process is shown in Figure 2.

### Data Collection and Analysis

Pharmacists provided aggregated, blinded data on the medication reviews and the PCPs’ responses in an Excel^®^ spreadsheet. Data were reported as follows:

- Patients’ demographic information: age, sex
- doMESTIC RISK assessment:^11^ review initiator, score obtained, missing information, time spent
- Medication review: availability of patient-related information, types and numbers of problems and/or unanswered questions identified, problems prioritised
- Communication with PCPs: types and numbers of suggestions/questions submitted
- Solution: types and numbers of suggestions accepted/questions answered; types and numbers of medication regimens changed
- Time spent

Patients’ identities were only known to their health care providers. Pharmacists assigned a study number to each patient containing the pharmacy’s project number followed by consecutive numbers for each patient included.

Data submitted by the participating pharmacies was combined in an Excel^®^ spreadsheet and analysed quantitatively in a descriptive form by two of the authors and verified by the others.

### Evaluation of Nurses’ and Pharmacists’ Experiences with the Project

The project concluded with an evaluation of home-care nurses’ and pharmacists’ opinions using written, profession-specific online questionnaires on the Findmind^®^ survey platform (www.findmind.ch). Due to the lack of validated questionnaires in this setting and for our target population, we used self-developed questionnaires focused on facilitators and barriers to interprofessional collaboration of this medication management program. The questionnaires were sent to all nurses and pharmacists involved in the study via snowball principle. Spitex Bern employed 237 nurses at the time of the study (www.spitex-bern.ch); it is unknown how many participated in the risk assessment and therefore, qualified for the evaluation.

Data analysis was performed directly in Findmind^®^ and partially exported to Excel^®^ and descriptively compiled.

We structured our report according to the SQUIRE 2.0 Standards for Quality Improvement Excellence.^12^

### Research Team

The research team consisted of four pharmacists (two community pharmacists and two clinical pharmacists), six pharmacy students from two different universities, two advanced practice nurses from the home-care setting and one senior physician (specializing in general internal medicine).

The study started on March 1, 2022 and was completed 6 months later, on August 31, 2022.

## Results

### Pharmacy Involvement

During recruitment, 14 community pharmacies in the city of Bern agreed to participate. However, during the six-month implementation phase, only ten calculated MRP risk scores and performed medication reviews. Of these ten pharmacies, eight (80%) submitted suggested interventions to PCPs. The complete results are detailed in Supplementary File D.

### Risk Assessment

*Spitex Bern* supported 436 patients with their medication management during the study period, 264 (61%) were eligible for inclusion. Of these, 106 patients (106/264, 40%) consented to participate, and the pharmacies returned 76 patient datasets (76/106, 72%). Pharmacists did not complete 30 risk tools; either patient-related information was missing and/or unobtainable via PCP or the patients were incorrectly allocated to pharmacies with which they had no existing customer relationship. One dataset was excluded due to ambiguity. In addition, 26/76 (33%) patients had a risk score of <5.

Forty-nine patients had a risk score of ≥5 and underwent a medication review by a pharmacist—34 (69%) women and 15 (31%) men. Their mean age was 84.0 ± 7.7 years and ranged from 65–103 years old.

Their mean risk score was 7.0 ± 2.1 points, ranging from 5–16 (maximum possible score: 17). All risk score calculations (100%) were initiated by a home-care nurse, none by the pharmacists. The time needed to complete the risk assessments was reported for 24/49 (49%) patients, with a mean of 13 ± 10 minutes, ranging from 5–45 minutes per professional group.

### Medication Reviews

Pharmacists completed 49 medication reviews. Patients were taking a mean of 11.2 ± 4.5 prescribed medications with a regular, repetitive dosage, ranging from 2–24 different medications, and a mean of 2.8 ± 3.5 prescribed as-needed medications, ranging from 0–14. Self-medication was rare, with a mean of 0.45 ± 1.0 medication per patient, ranging from 0–4 medication(s).

Although pharmacists mostly had access to complete medication lists, much other clinical information was missing. Their access to clinical data is shown in Table 1.

**Table 1:** Clinical Data Available to Pharmacists During Medication Reviews

Type of Clinical Data	Availability n = patients, patients in %
Complete medication list	48/49, 98%
Partial medication list*	15/49, 31%
List of diagnoses	26/49, 53%
Laboratory values	6/49, 12%
Estimated glomerular filtration rate eGFR	13/49, 27%
Vital signs	5/49, 10%

**Notes**: *Physicians did not provide a comprehensive list of current medications, but just information on recently adapted or newly added medication.

The time needed to complete medication reviews was reported in 42/49 cases (86%), with a mean of 40 ± 24 minutes, ranging from 10–120 minutes.

In the 49 medication reviews completed by the ten pharmacies, pharmacists identified 120 potential MRPs in 31/49 (63%) patients. No MRPs were identified among the other 18 patients (18/49, 37%).

For 13 patients (13/31, 42%), the potential MRPs identified by pharmacists were deemed non-priority by pharmacists and therefore, not forwarded to PCPs. This resulted in a total of 93 MRPs identified as relevant for 18 patients (mean 5 MRPs/patient, median 2, ranging from 1–41). An overview of the MRPs is shown in Table 2.

**Table 2:** Potential MRPs Identified by Community Pharmacists During Structured Medication Reviews

Types of Potential MRPs	Potential MRPs Identified by Pharmacists (n)	Potential MRPs Assessed as Requiring an Intervention (n, (%))
Contraindication: relative	27	19 (15.6%)
PIMs/Priscus^®^	14	10 (8.3%)
Unsuitable time/frequency of administration	11	8 (6.7%)
Overdose	10	10 (8.3%)
Clinically relevant interaction	9	7 (5.8%)
Therapy not according to current guidelines	9	6 (5.0%)
Inappropriate duration of treatment	8	8 (6.7%)
Overtreatment	7	3 (2.5%)
Therapy not indicated	7	6 (5.0%)
Undersupply	6	6 (5.0%)
Unsuitable route/form of administration	5	3 (2.5%)
Lack of monitoring	4	4 (3.3%)
Contraindication: absolute	2	2 (1.7%)
Other	1	1 (0.8%)
Underdosing	0	0
Duplication	0	0
Total MRPs	120	93/120 (77.5%)
**Total relevant MRPs n (%)**	**93/120 (77.5%)**	

**Notes**: Priscus list: www.priscus2-0.de. Relative contraindication: a signal that a drug is generally discouraged due to increased risk, but may be considered if the potential benefits outweigh the risks and if precautionary measures are applied. Absolute contraindication: the drug must be strictly avoided under all circumstances due to the risk of life-threatening or severe harm.

**Abbreviation**: MRP, medication-related problem; PIM, potentially inappropriate medication.

The 93 potentially relevant MRPs identified by pharmacists led to the derivation of 64 potential interventions. Of those, 43/64 (67.2%) interventions for 18/49 (36.7%) patients were assessed to be priorities and communicated to PCPs. An overview of the interventions is displayed in Table 3.

**Table 3:** Summary of Pharmacists’ Intervention Suggestions, Response Rates from Primary Care Physicians (PCPs), and Final Acceptance Rates

Type of Intervention	Potential Interventions Identified [n]	Priority Interventions Communicated to PCPs [n, % Interventions Identified]	Interventions Responded to by PCPs [n, % Priority Interventions]	Suggested Interventions Accepted by PCPs [n, % Interventions Responded to and Accepted]
Total interventions	64	43, 67.2%	22, 51.2%	14, 63.6%
Deprescribing - dose reduction	17, 26.6%	9, 52.9%	7, 77.8%	7, 100.0%
Deprescribing—therapy cessation	15, 23.4%	8, 53.3%	2, 25.0%	2, 100.0%
Generic substitution	4, 6.3%	0, 0.0%	n/a	n/a
Therapeutic substitution	3, 4.7%	3, 100.0%	2, 25.0%	0, 0%
Therapeutic monitoring	6, 9.4%	2, 33.3%	3, 66.7%	1, 33.3%
Changing administration time	6, 9.4%	4, 66.7%	2, 50.0%	2, 100.0%
Therapy simplification	5, 7.8%	5, 100.0%	0, 0.0%	n/a
Therapy initiation	5, 7.8%	4, 80.0%	3, 75.0%	0, 0.0%
Other	3, 4.7%	3, 100.0%	5, 62.5%	2, 40.0%
Unknown	n/a	5, n/a		
Dose increase	0, 0.0%	n/a	n/a	n/a

**Abbreviations**: PCP, primary care physician; n/a, not applicable.

In cases where with a suggested intervention to PCPs, the pharmacists had more clinical information available: a medication list in 100% of cases (18/18) vs 98% in the overall study population, a diagnoses list in 83% vs 53%, and laboratory values in 22% vs 12%, respectively.

Despite being provided with comprehensive information on patients current status and interventions suggested, PCPs only responded to pharmacists for 8/18 patients (44.4%). The remaining attempts (10/18 patients, 55.6%) to establish contact remained unanswered. The most responses addressed deprescribing (77.8%), with 100% of those suggestions being accepted. The second category most frequently responded to was therapy initiation (75.0%); however, 100% of these suggestions were declined. Overall, 63.3% of the suggested interventions responded to were accepted. Details are shown in Table 3.

Pharmacists also implemented therapy changes of their own accord, informing PCPs afterwards in 15.0% (2/13) of those interventions. The interventions were generic substitution (8 times), changing administration times (twice), restarting a medication (twice) and monitoring (once).

### Evaluation

Of 14 participating pharmacies, ten (71%) completed the survey, as did 12 home-care nurses. On a Likert scale from 1 (very good) to 5 (very bad), nurses rated their patients’ medication safety as mediocre (mean 2.8 ± 0.8), with 91.7% of them (11/12) seeing at least some potential for optimisation.

When collaborating with community pharmacies, home-care nurses reported that they would like more support in organising prescriptions and more proactive direct collaboration between pharmacists and PCPs when prescription clarification was needed. When home-care nurses collaborated with PCPs, missing (current) medication lists was their most pressing issue.

Half of the participants (50%) considered regular use of a risk assessment tool like doMESTIC RISK^11^ to be problematic due to limited time and personnel. Pharmacists were amenable—if backed by additional resources and with billable hours—to taking on quality improvement tasks and initiating risk assessments. They also saw a clear role allocation between pharmacists, home-care nurses and PCPs as necessary for future medication reviews.

One-third of pharmacists considered their home-care patients’ medication safety to be mediocre; none considered it bad or very bad. Nevertheless, 63% of them saw the potential for optimisation, with communication about current medication lists especially lacking. Most participants (88%) considered structured processes as beneficial to interprofessional collaboration, but not necessarily with PCPs due to their lack of acceptance of pharmacists’ new roles and the services. At the same time, half of participating pharmacists (50%) considered the doMESTIC RISK assessment tool^11^ too time-consuming relative to its benefits. Pharmacists would prefer nurses to initiate the process. Although 63% of pharmacists envisioned conducting medication reviews in the future, they also need additional resources and reimbursement solutions. The lack of communication channels between health care professionals was considered a major obstacle. Pharmacists were worried that they were not generally considered part of patients’ health care teams, which might further impede the exchange of clinical data under Switzerland’s current data protection act. This might also hamper interprofessional assessment when using the doMESTIC RISK tool. In addition, the pharmacists reported that the term “medication review” was ambiguously defined. Depending on the depth of the medication review performed, pharmacists might need additional clinical education. What pharmacists appreciated most about the present study was the opportunity for interprofessional collaboration with home-care nurses, getting to know home-care’s organisational structure and needs better, and deepening their knowledge on how to perform medication reviews.

## Discussion

The burdens of polypharmacy and potentially inappropriate medications, especially among vulnerable older adults, are an important medication safety and, ultimately, a patient safety issue.

In a previous review we conducted, home-care patients were prescribed between 5 and 14 medications daily.^6^ The Helsana drug utilization report, analyzing claims data from 2019, stated that 87.3% of home-care clients had at least five different medications, the definition of polypharmacy in this analysis.^8^ The prevalence of PIMs ranged between 19.8% and 48.4%, with up to 26% classified as severe in our review.^6^ Helsana reported that at least one medication that is not suitable for older people, according to the Beers Criteria and/or the Priscus list, was prescribed to 71.1% of all Spitex clients.^8^ A number much higher than reported in our review, which might be due to differing patient characteristics in different home-care organizations.

Thus, they are a significant target group for medication safety interventions.^6^

The present study assessed whether an intervention to optimise interprofessional collaboration could improve the medication regimens of home-care patients, with a focus on deprescription.

Our study did not alter the customary professional structures of ambulatory health care in Switzerland but tried to prioritise patients at risk of MRPs and improve interprofessional collaboration. The ultimate goal was to assess the usefulness of the process and materials envisioned in the study’s blueprint. Several obstacles were evident in our findings. While the study demonstrated that home-care nurses and pharmacists could jointly prioritise patients in need of a medication review, actively recruiting patients consenting to such a review proved challenging. Indeed, many patients potentially benefitting from a medication review missed out. Because up to 65% of home-care patients can be housebound^13^ and unable to visit a community pharmacy, concerted efforts may prove necessary to identify those patients who would benefit the most.

Overall, two-thirds of the patients assessed had a risk score qualifying them for a medication review. This demonstrated that, despite a high prevalence of polypharmacy, prioritisation is possible. In light of the limited resources and reimbursement solutions, targeting clinical pharmacy services to the home-care patients most at risk of MRPs could be an efficient approach, as described in other settings.^14^,^15^

Up-to-date, complete, accurate and timely medication information is crucial to ensuring medication safety.^9^ In our analysis, community pharmacists have very limited access to patients’ clinical information. Even lists of diagnoses are often missing, limiting the scope of medication reviews, according to the Pharmaceutical Care Network Europe classification.^16^ This might have contributed to the very short duration of some of the individual medication reviews reported. Limited clinical information might negatively affect the pertinence of medication reviews. This might also have played a role in the limited number of physicians’ responses. On the other hand, PCPs accepted almost 63.0% of the pharmacists’ suggestions. This was comparable to a similar study in Belgium, which reported an acceptance rate of almost 68%.^17^ However, one weakness of our study’s design was the information-only approach to integrating PCPs into the workflow. In the future, medication reviews should be embedded into an organisational process designating proactive roles for all professionals involved. This was also reflected in a recent study that identified the development of a culture of interprofessional cooperation as a central factor in the success of pharmaceutical care, alongside time and a setting creating opportunities to interact.^18^

Pharmacists identified potential MRPs in two-thirds of patients eligible for a medication review, with a median of two MRPs per patient. This was comparable to a recent study that reported 2.6 MRPs per patient in ambulatory care.^19^

Within the scope of our study, pharmacists were instructed to prioritise prescription interventions for discussion with PCPs. This led to a limitation in the number of interventions suggested by pharmacists during their initial contacts with PCPs, as intended. The study’s goal of focusing on deprescribing was also met: deprescribing was the most frequently suggested category of interventions and had an optimal acceptance rate of 100%. In the study by Vink et al, discontinuation of a drug was also the most commonly suggested intervention by pharmacists, with an acceptance rate of 64%, comparable to ours.^20^

Nevertheless, more direct feedback would facilitate the definition of common interprofessional goals for individual patients’ health and a mutual understanding of different professionals’ respective roles in the health care team. Interprofessional quality circles and case study discussions might be a way forward.^21^,^22^ These efforts might also optimise information flow, as described in a similar setting.^23^ In the study published by Vink et al in 2011, physician response was comparably limited, amounting to 63% of requests answered. Communication across different settings was also identified as a major barrier to interprofessional collaboration in the scope of medication reviews.^20^

In the future, it might also be of interest to assess the preventability of adverse medication-related effects identified by pharmacists.^24^ A future longitudinal cohort study would also allow for the assessment of the lasting effects of implemented interventions and cost savings not only due to deprescribing but also in order to estimate a potential reduction in morbidity, rehospitalisations and mortality.

### Limitations

Our study had some limitations. Patient recruitment was done by home-care nurses exclusively. They may have recruited patients due to specific characteristics, such as a willingness to participate, potentially leading to selection bias. Nevertheless, patient agreement is crucial to optimising medication therapy, and shared decision-making is a cornerstone of successful therapy, specifically via its influence on adherence in multimorbid, polymedicated patients.^25^

Our findings pertaining to medication optimisation centered predominantly around pharmacists’ abilities to perform high-quality medication reviews. The quality of their findings might, therefore, have influenced PCPs’ acceptance rate. Although clinical pharmacy is now an integral part of pharmacy education across Switzerland, this was not necessarily the case when older colleagues graduated. To ensure the quality of medication reviews, targeted continuing education programs might empower pharmacists.

Many cantons in Switzerland allow physician dispensing—the direct distribution of medications to patients in a PCP’s practice. In the canton of Bern, our study setting, physicians’ offices at a certain distance from a pharmacy are allowed to dispense medications directly to their patients. This might have hampered the collaborative behaviour between PCPs and pharmacists, as different studies have detailed.^26^

The number of patients finally included in this study was very limited and might in turn limit the generalizability of our findings. Especially in light of the limited interprofessional collaboration detailed above.

Last but not least, reimbursement for the coordination of care and interprofessional collaboration is lacking in Switzerland’s act-based professional reimbursement scheme. This may also have influenced participants’ opportunities and willingness to collaborate interprofessionally.

## Conclusions

Within the scope of a structured medication management program involving a risk assessment tool for prioritising older adult home-care patients, community pharmacists, in collaboration with home-care nurses and primary care physicians, identified a variety of aspects with the potential to improve medication therapy through medication review: most prominently deprescribing through dose reduction and therapy cessation. However, there were several barriers to that program’s successful implementation: foremost, a lack of clinical information accessible to all health care professionals, potentially limiting the pertinence of the prescription interventions the pharmacists suggested. Optimising means of communicating information and creating platforms for professional exchanges also seem necessary. Means of reimbursing health care professionals in Switzerland for their coordination activities and interprofessional collaboration are almost non-existent under the country’s act-based payment system. When implementing interprofessional medication management programs, shared decision-making about role allocation is crucial to a program’s success. In addition, the implementation of a tool used to prioritize patients at risk for collaborative services might allocate resources in a more meaningful and targeted way. In the scope of a larger study, statistical analysis would allow for more information on the impact of interprofessional medication reviews on the medication safety of home-care patients.

## Impact of Findings on Practice Statements

- doMESTIC RISK – a 10-item risk assessment tool – was feasible for use in home care nurses and pharmacists clinical practice in order to prioritize patients at risk for medication reviews.
- Community pharmacists, in collaboration with home-care nurses and primary care physicians, identified a variety of aspects with the potential to improve medication therapy in the scope of structured medication reviews.
- Access to clinical information for all health care professionals involved was a significant barrier to the performance of relevant medication reviews.
- Means of reimbursing health care professionals for their coordination activities and interprofessional collaboration are crucial for the success of future programmes.
